# Association of metabolic syndrome and its components with arterial stiffness in Caucasian subjects of the MARK study: a cross-sectional trial

**DOI:** 10.1186/s12933-016-0465-7

**Published:** 2016-10-24

**Authors:** Leticia Gomez-Sanchez, Luis Garcia-Ortiz, M. Carmen Patino-Alonso, Jose I. Recio-Rodriguez, Rigo Fernando, Ruth Marti, Cristina Agudo-Conde, Emiliano Rodriguez-Sanchez, Jose A. Maderuelo-Fernandez, Rafel Ramos, Manuel A. Gomez-Marcos, the MARK Group

**Affiliations:** 1 Primary Care Research Unit, The Alamedilla Health Center, 37003 Salamanca, Spain; 2 Castilla and León Health Service (SACyL), Biomedical Research Institute of Salamanca (IBSAL), Salamanca, Spain; 3 Biomedical and Diagnostic Sciences Department, University of Salamanca, Salamanca, Spain; 4 Statistics Department, University of Salamanca, Salamanca, Spain; 5 San Agustín Health Center, Illes Balears Health Service (IBSALUT), Palma of Mallorca, Spain; 6 Unitat of Suport a the Recerca of Girona, Institut Universitari D’Investigació in Atenció Primària Jordi Gol (IDIAP Jordi Gol), Girona, Spain; 7 Institut d’Investigació Biomèdica de Girona Dr. Josep Trueta (IDBGI), Girona, Spain; 8 Department of Medicine, University of Salamanca, Salamanca, Spain; 9 Departament of Ciències Mèdiques, Facultat de Medicina, Universitat de Girona, Girona, Spain; 10 MARK Group. RedIAPP: Research Network in Preventive Activities and Health Promotion, Girona, Spain

**Keywords:** Metabolic syndrome, Brachial-ankle pulse wave velocity, Cardio-ankle vascular index, Arterial stiffness

## Abstract

**Background:**

The cardio-ankle vascular index (CAVI) and brachial-ankle pulse wave velocity (baPWV) can reflect both central and peripheral arterial stiffness. Metabolic syndrome (MetS) and its components may increase arterial stiffness and the risk of cardiovascular diseases. However, the correlation of MetS and its components with arterial stiffness is still not clear. The primary aim of this study is thus the relationship using baPWV and CAVI in Caucasian adults with intermediate cardiovascular risk. The secondary aim is to analyze sex differences.

**Methods:**

This study analyzed 2351 subjects aged 35–74 years (mean, 61.4 ± 7.7 years) comprising 61.7 % males and enrolled in the *improving interMediAte Risk management (MARK)* study. CAVI was measured using a *VaSera VS*-*1500* ^*®*^ device, and baPWV was calculated using a validated equation. MetS was defined based on the Joint Scientific Statement National Cholesterol Education Program III. Waist circumference, blood pressure, fasting plasma glucose, and lipid profile were measured.

**Results:**

MetS was found in 51.9 % of the subjects. All MetS components except reduced HDL-cholesterol (p = 0.578) were associated with CAVI. High density lipoprotein cholesterol (p = 0.075) and waist circumference (p = 0.315) were associated with baPWV. The different MetS components that assess dyslipidemia using the stiffness measures show different associations according to patient sex. The high blood pressure component had a greater odds ratio (OR) for both baPWV ≥ 17.5 m/sec (OR = 6.90, 95 % CI 3.52–13.519) and CAVI ≥ 9 (OR = 2.20, 95 % CI 1.63–1.90).

**Conclusions:**

MetS and all its components (except HDL-cholesterol with baPWV and CAVI and WC with baPWV) were associated with baPWV and CAVI. However, there were sex differences in the association of MetS and its components with baPWV and CAVI. Data from this study suggest a greater association of CAVI and baPWV values with MetS components in males than in females and indicate greater arterial stiffness in the event of simultaneously elevated blood pressure, fasting plasma glucose, and waist circumference.

*Trial Registration* Clinical Trials.gov Identifier: https://clinicaltrials.gov/ct2/show/ NCT01428934. Registered 2 September 2011. Last updated September 8, 2016

**Electronic supplementary material:**

The online version of this article (doi:10.1186/s12933-016-0465-7) contains supplementary material, which is available to authorized users.

## Background

Metabolic syndrome (MetS) is a cluster of multiple risk factors for atherosclerosis that include obesity, high blood pressure, elevated fasting plasma glucose (FPG), and atherogenic dyslipidemia [1]. MetS doubles the risk of morbidity and mortality from cardiovascular diseases and multiplies the risk of all-cause mortality by 1.5 [2–4]. However, the risk varies according to the associated components on which the MetS diagnosis is based [5]. For example, in the Framingham Heart Study cohort, the combination of central obesity, blood pressure (BP), and FPG increased the risk of mortality three-fold [6]. Similarly, different combinations of MetS components have different effects on arterial stiffness [7–9]. Several studies have also reported that greater arterial stiffness is associated with increased morbidity and mortality from cardiovascular diseases [10–12].

Arterial stiffness may be evaluated using the brachial-ankle pulse wave velocity (baPWV) [13] and the cardio-ankle vascular index (CAVI) [14]. baPWV has been demonstrated as an independent predictor of coronary artery disease and all-cause mortality in general populations and in subjects with diabetes [15]. CAVI is a measure [14] of overall arterial stiffness starting from the aorta all the way to the ankle [16]. It is associated with carotid and coronary atherosclerosis [10, 17–19] and is a predictor of cardiovascular events in the obese [10]. CAVI is a better predictor of coronary artery disease than baPWV [20, 21].

Studies have investigated the relationship of MetS and its components with baPWV [7, 22–24]. Most have reported increased baPWV in subjects with MetS or with a greater number of MetS components. Most of the studies have examined Asian populations, among whom MetS and its components affect arterial stiffness with greater severity in females than in males [23, 25]. It is likely that females with MetS develop more severe atherosclerosis, but this is not yet definitive [25]. Furthermore, the sex-dependent association of the specific cluster of MetS components with baPWV and CAVI in Caucasian adults with intermediate cardiovascular risk has not been previously.

Research on the relationship of MetS and its components with baPWV and CAVI is an important topic around the world. Unlike baPWV, the characteristics of CAVI as a physiological marker of arterial stiffness have not frequently been reported. The primary aim of this study is to investigate the relationship of MetS and its components with arterial stiffness measured by baPWV and CAVI in Caucasian adults with intermediate cardiovascular risk, with the secondary aim of analyzing sex differences.

## Methods

### Study design

This trial is a cross-sectional study of subjects recruited to the *improving interMediAte RisK management (MARK)* study (NCT01428934) [26], which is a longitudinal study designed to assess whether the ankle-brachial index, arterial stiffness (measured by CAVI), postprandial glucose, glycosylated hemoglobin, self-measured blood pressure, and the presence of comorbidities are independently associated with the occurrence of vascular events. It also investigates whether the predictive capacity of current risk equations can be improved in the intermediate risk population. The current study focuses on the baseline visit. The second step will be a 5- and 10 year follow-up trial to assess cardiovascular morbidity and mortality.

### Study population

In this multicenter project, sample selection was performed by random sampling from among individuals who met the inclusion criteria and were seeing general practitioners at six primary care centers in three Spanish Autonomous Communities from July 2011 to June 2013. Subjects were recruited from those aged 35 to 74 years with intermediate cardiovascular risk defined as 10 year coronary risk ranging from 5–15 % according to the adapted Framingham risk equation [27]; 10 year vascular mortality risk ranging from 1–5 % according to the scoring risk in Europeans equation [28]; or moderate risk according to the European Society of Hypertension guidelines for the management of arterial hypertension [29]. The analysis examined 2351 of the 2495 subjects recruited to the MARK study. Exclusion criteria included end-stage disease or institutionalization at the time of the visit, or a history of atherosclerotic disease. Subjects were excluded for the following reasons: 88 had an altered ankle-brachial index, measured CAVI and/or baPWV values were not available for 23 subjects, and MetS components had not been measured for 33 subjects. The study was approved by the Research Ethics Committees of the Primary Care Research Institute of Jordi Gol, Health Care Area of Salamanca and Palma of Mallorca. All participants gave written informed consent according to the general recommendations of the Declaration of Helsinki [30].

### Variables and measurement instruments

A detailed description of procedures for clinical data collection, anthropometric measurements, and laboratory tests has been published elsewhere [26].

### Diagnostic criteria of MetS

According to the international consensus in the Joint Scientific Statement National Cholesterol Education Program III [1], MetS was defined as the presence of three or more of the following five components: abdominal obesity (waist circumference (WC) ≥88 cm in females and ≥102 cm in males); elevated triglycerides (TGC) ≥150 mg/dL (or drug treatment for elevated TGC); high-density lipoprotein (HDL) cholesterol <40 mg/dL in males or <50 mg/dL in females; high blood pressure (systolic blood pressure (SBP) ≥130 mmHg or diastolic blood pressure (DBP) ≥85 mmHg, or antihypertensive drug treatment), and fasting plasma glucose (FPG) ≥100 mg/dL (or drug treatment for elevated glucose).

### Analysis groups

In order to analyze the influence of the different combinations of MetS components upon arterial stiffness, the subjects were divided into three groups. The group “MetS-dyslipidemia” consisted of 367 subjects with components of high blood pressure and those that indicate dyslipidemia (low HDL-cholesterol and elevated triglycerides). The group “MetS-increased insulin resistance” consisted of 511 subjects with the components of high blood pressure and those that indicate increased insulin resistance (elevated FPG and abdominal obesity). The group “MetS-mixed” consisted of 342 subjects with MetS who were not included in either the dyslipidemia group or the increased insulin resistance group. The control group consisted of 175 subjects without MetS, arterial hypertension, FPG, or the use of antihypertensive, lipid-lowering, or antidiabetic drugs.

### Diagnosis of cardiovascular risk factors

Subjects were considered hypertensive if previously diagnosed with hypertension, if they were taking antihypertensive drugs, or if they had blood pressure levels ≥140/90 mmHg. Diabetic subjects were those who had a previous diagnosed of the disease, were taking hypoglycemic drugs, or had fasting blood glucose levels ≥126 mg/dL or HbA1c ≥6.5 %. Dyslipidemia was defined as a prior diagnosis of the condition, use of lipid-lowering drugs, or fasting total cholesterol levels ≥250 mg/dL.

### Cardio-ankle vascular index (CAVI) and brachial-ankle pulse wave velocity (baPWV)

CAVI was measured using a *VaSera VS*-*1500* ^*®*^ device *(Fukuda Denshi*) [31, 32]. CAVI values are calculated automatically by estimating the stiffness parameter β with the following equation: β = 2ρ × 1/(Ps−Pd) × ln (Ps/Pd) × PWV^2^, where ρ is blood density, Ps and Pd are SBP and DBP in mmHg, and PWV is measured between the aortic valve and the ankle [14]. The mean coefficient of variation of CAVI measurement is less than 5 %, which is small enough to allow for clinical use of the index and confirms that CAVI has a favorable reproducibility [32].

baPWV was estimated using the equation, baPWV = (0.5934 × height (cm) + 14.4724)/tba (tba is the time interval between the arm and ankle waves) [13]. Measurements were performed with the patient in supine position after resting for 10 min in a quiet room at a stable temperature. Subjects were instructed not to smoke or practice exercise in the hour prior to the test.

CAVI was classified as normal (CAVI < 8), borderline (8 ≤ CAVI < 9), or abnormal (CAVI ≥ 9). Abnormal CAVI represents subclinical atherosclerosis [ 14, 33–36 ]. A value of baPWV ≥17.5 m/sec was considered abnormal [37]. The average values of CAVI and baPWV were considered.

### Office or clinical blood pressure

Office blood pressure measurement involved three measurements of SBP and DBP with a validated OMRON model M10-IT sphygmomanometer (Omron Health Care, Kyoto, Japan). The measurements followed the recommendations of the European Society of Hypertension [38], and the averages of the last two measurements used.

### Anthropometric measurements

Body weight was measured twice with a certified electronic scale (Seca 770, Medical scale and measurement systems, Birmingham, United Kingdom) after adequate calibration (precision ±0.1 kg). Readings were rounded to 100 g. Height was measured with a stadiometer (Seca 222), and the average of two measurements was recorded. Body mass index (BMI) was calculated as weight (kg) divided by height squared (m^2^). Waist circumference was measured according to the 2007 recommendations of the Spanish Society for the Study of Obesity [39]. All measurements were performed with the subjects standing, wearing no shoes, and in light clothing. The researchers who performed the different tests were blinded to the clinical data of the subjects. All assessments were made within a period of 10 days.

### Statistical analysis

Continuous variables are expressed as the mean ± standard deviation. Categorical variables are presented as frequency distributions and compared using the Chi-squared test or Fisher’s exact test when necessary. The difference in means between 2-category and quantitative variables was analyzed using the student’s t test for independent samples. ANCOVA models were used to test the differences in mean baPWV and CAVI values with the five components of MetS. Pairwise post hoc comparisons were examined using the Bonferroni test.

In the multivariate analysis, six multiple linear regression models were performed (ENTER method) using CAVI and baPWV as dependent variables and MetS and its components as independent variables of each model. Six logistic regression models were also developed in which baPWV (<17.5 = 0 and ≥17.5 = 1) and CAVI status (<9 = 0 and ≥9 = 1) were dependent variables, while the independent variables were the absence (0) or presence (1) of MetS and its components. All models included age, height, weight, antihypertensive drugs, lipid-lowering drugs, and antidiabetic drugs as adjusting variables. The exception was WC, which was adjusted for age and drug use because of collinearity problems. Analyses were performed for the subjects overall and according to gender. Data were analyzed using SPSS Statistics for Windows version 23.0 (IBM Corp, Armonk, NY). Values of p < 0.05 were considered statistically significant.

## Results

### Clinical characteristics of all subjects

Table 1 shows the characteristics (overall and by sex) of the 2351 subjects analyzed. Mean age was 61.4 ± 7.7 years. MetS was found in 51.9 % of the subjects (46.0 % of males and 61.4 % of females). The mean baPWV was 14.9 ± 2.6 m/sec (coefficient of variation: 0.172), and the mean CAVI was 8.8 ± 1.2 (coefficient of variation: 0.133). CAVI was higher among males than females (8.9 ± 1.2 versus 8.6 ± 1.1; p < 0.001). Mean baPWV values were similar among both sexes.

**Table 1:** Characteristics of subjects global and stratified by gender

Variables	Global (n = 2351)	Males (n = 1450)	Females (n = 901)	p value
Age, (years)	61.4 ± 7.7	61.2 ± 8.1	61.8 ± 7.0	0.044
Smoking, n (%)	659 (28.1)	455 (31.4)	204 (22.7)	<0.001
BMI, (kg/m^2^)	29.2 ± 4.4	29.1 ± 3.9	29.5 ± 5.1	0.016
BMI ≥ 30, n (%)	850 (36.2)	485 (33.4)	365 (40.5)	0.001
WC, (cm)	100.9 ± 11.6	102.9 ± 10.5	97.6 ± 12.5	<0.001
SBP, (mmHg)	137.0 ± 17.4	138.9 ± 17.1	134.1 ± 17.5	<0.001
DBP, (mmHg)	84.4 ± 10.2	85.5 ± 10.3	82.6 ± 9.6	<0.001
Hypertension, n (%)	1842 (78.3)	1163 (80.2)	679 (75.4)	0.006
Antihypertensive drugs, n (%)	1208 (51.4)	733 (50.6)	475 (52.7)	0.309
Total Cholesterol, (mg/dl)	225.7 ± 41.0	220.6 ± 38.0	233.9 ± 42.8	<0.001
LDL-C, (mg/dl)	140.4 ± 34.9	138.9 ± 34.2	142.7 ± 35.8	0.010
HDL Cholesterol, (mg/dl)	49.8 ± 12.9	47.9 ± 11.9	52.9 ± 13.8	<0.001
TGC, (mg/dl)	145.7 ± 97.0	150.5 ± 106.7	138.0 ± 78.2	0.002
Dyslipidemia, n (%)	1583 (67.3)	924 (63.7)	659 (73.1)	<0.001
Lipid lowering drugs, n (%)	673 (28.6)	392 (27.0)	281 (31.2)	0.031
FPG, (mg/dl)	107.7 ± 34.4	107.4 ± 33.5	108.0 ± 35.9	0.711
HA1c, (%)	6.1 ± 1.2	6.1 ± 1.1	6.2 ± 1.3	0.001
Diabetes mellitus type 2, n (%)	794 (33.8)	464 (32.0)	330 (36.6)	0.022
Antidiabetic drugs, n (%)	477 (20.3)	270 (18.6)	207 (23.0)	0.015
Higher blood pressure, n (%)	1986 (84.5)	1253 (86.4)	733 (81.4)	0.001
Higher FPG, n (%)	1112 (47.3)	693 (47.8)	419 (46.5)	0.552
Lower HDL-C, n (%)	791(33.6)	357 (24.6)	434 (48.2)	<0.001
Higher TGC, n (%)	839 (35.7)	545 (37.6)	294 (32.6)	0.015
Higher WC, n (%)	1478 (62.9)	762 (52.6)	716 (79.5)	<0.001
Metabolic syndrome, n (%)	1220 (51.9)	667 (46.0)	553 (61.4)	<0.001
CAVI	8.80 ± 1.17	8.91 ± 1.19	8.65 ± 1.12	<0.001
CAVI ≥ 9, n (%)	1061 (45.1)	701 (49.6)	360 (41.3)	0.001
baPWV, (m/s)	14.87 ± 2.57	14.81 ± 2.56	14.98 ± 2.59	0.119
baPWV ≥ 17.50 m/s, n (%)	324 (13.8)	192 (13.2)	132 (13.9)	0.356

Values are means (standard deviations (SD) for continuous data and number and proportions for categorical data

Metabolic syndrome: Three or more of the following criteria: (Abdominal obesity = Higher WC: waist circumference ≥88 in females and ≥102 in males. Higher blood pressure: SBP ≥130 mmHg and/or DBP ≥85 mmHg or antihypertensive drug treatment. Higher FPG: FPG >100 mg/dl or antidiabetic drug treatment. Lower HDL cholesterol: HDL cholesterol <40 mg/dl in males and <50 mg/dl in females. Higher triglycerides: TGC >150 mg/dl)

*BMI* body mass index; *WC* waist circumference; *SBP* systolic blood pressure; *DBP* diastolic blood pressure; *LDL-C* low density lipoprotein cholesterol; *HDL-C* high density lipoprotein cholesterol; *TGC* triglycerides; *FPG* fasting plasma glucose; *HbA1c* glycosylated hemoglobin; *CAVI* cardio-ankle vascular index; *baPWV* brachial-ankle pulse wave velocity

p value differences in male and females

Table 2 shows differences between subjects with and without MetS by sex. baPWV was higher in subjects with MetS, regardless of sex. Mean CAVI values were not different between subjects with and without MetS in either sex.

**Table 2:** Characteristics of subjects stratified by gender and the presence/absence of metabolic syndrome

Variables	Males subjects (n = 1450)		Females subjects (n = 901)	
	MetS + n = 667	MetS − n = 783	MetS + n = 553	MetS − n = 348
Age, (years)*	60.5 ± 8.1	61.8 ± 8.1	61.7 ± 7.1	62.1 ± 6.9
SBP, (mmHg)*^†^	141.4 ± 16.1	136.7 ± 17.5	136.3 ± 17.1	130.6 ± 17.7
DBP. (mmHg)*^†^	87.5 ± 10.3	83.7 ± 10.1	83.5 ± 9.2	81.2 ± 10.2
Hypertension, n (%)*^†^	600 (90.0)	563 (71.9)	469 (84.8)	210 (60.3)
Antihypertensive drugs, n (%)*^†^	404 (60.6)	329 (42.0)	352 (63.7)	123 (35.3)
HDL-C, (mg/dl)*^†^	43.2 ± 10.3	51.9 ± 11.8	48.3 ± 11.2	60.3 ± 14.2
TGC, (mg/dl)*^†^	184.0 ± 132.4	116.8 ± 54.0	160.8 ± 87.9	101.8 ± 37.5
Dyslipidemia, n (%)	436 (65.4)	488 (62.3)	403 (72.9)	256 (73.6)
Lipid lowering drugs, n (%)*^†^	207 (31.0)	185 (23.6)	197 (35.6)	84 (24.1)
FPG (mg/dl)*^†^	118.6 ± 37.9	98.0 ± 25.8	118.2 ± 39.3	91.8 ± 21.3
Diabetes mellitus type 2, n (%)*^†^	329 (49.3)	135 (17.2)	289 (52.3)	41 (11.8)
Antidiabetic drugs, n (%)*^†^	198 (29.7)	72 (9.2)	188 (34.0)	19 (5.5)
WC, (cm)*^†^	108.3 ± 10.1	98.3 ± 8.5	101.5 ± 11.3	91.4 ± 11.6
Higher blood pressure, n (%)*^†^	644 (96.6)	609 (77.8)	512 (92.6)	221 (63.5)
Higher FPG, n (%)*^†^	494 (74.1)	199 (25.4)	378 (68.4)	41 (11.8)
Lower HDL-C, n (%)*^†^	295 (44.2)	62 (7.9)	372 (67.3)	62 (17.8)
Higher TGC, n (%)*^†^	423 (63.4)	122 (15.6)	269 (48.6)	25 (7.2)
Higher WC, n (%)*^†^	539 (80.8)	223 (28.5)	516 (93.3)	200 (57.5)
*CAVI*	*8.86 ± 1.24*	*8.94 ± 1.14*	*8.66 ± 1.19*	*8.61 ± 1.01*
*CAVI ≥ 9, n (%)*	*319 (49.3)*	*382 (49.9)*	*230 (42.8)*	*130 (38.9)*
baPWV, (m/s)*^†^	*14.98 ± 2.50*	*14.66 ± 2.61*	*15.23 ± 2.59*	*14.60 ± 2.36*
*baPWV ≥ 17.5 m/s, n* (%)	*94 (14.1)*	*98 (12.5)*	*97 (17.5)*	*35 (10.1)*

Values are means (standard deviations (SD) for continuous data and number and proportions for categorical data

Metabolic syndrome: Three or more of the following criteria: (abdominal obesity = higher WC: waist circumference ≥88 in females and ≥102 in males. Higher blood pressure: SBP ≥130 mmHg and/or DBP ≥85 mmHg or antihypertensive drug treatment. Higher FPG: FPG >100 mg/dl or antidiabetic drug treatment. Lower HDL cholesterol: HDL cholesterol <40 mg/dl in males and <50 mg/dl in females. Higher triglycerides: TGC >150 mg/dl)

*MetS* metabolic syndrome; *SBP* systolic blood pressure; *DBP* diastolic blood pressure; *HDL-C* high density lipoprotein cholesterol; *TGC* triglycerides; *FPG* fasting plasma glucose; *WC* waist circumference; *CAVI* cardio-ankle vascular index; *baPWV* brachial-ankle pulse wave velocity

* p < 0.05 in males, ^†^ p < 0.05 in females

### Association between the MetS and its components with arterial stiffness

After adjustment for potentially influencing variables, a multiple linear regression analysis showed that almost all MetS components were associated with baPWV and CAVI except for reduced HDL-cholesterol with respect to CAVI (p = 0.578) and baPWV (p = 0.075) and WC with respect to baPWV (p = 0.315). Among males, all MetS components (except HDL-cholesterol with respect to CAVI and WC with respect to baPWV) were associated with the two arterial stiffness measures. However, among females, SBP, DBP, and FPG were associated with baPWV and CAVI, and WC was associated with CAVI (Table 3).

**Table 3:** Associations of MetS components with baPWV and CAVI values global and by gender

Components MS	Global (n = 2351)			Males subjects (n = 1450)			Females subjects (n = 901)		
	β (95 % CI)	R^2^	p value	β (95 % CI)	R^2^	p value	β (95 % CI)	R^2^	p value
*Dependent variable: baPWV*									
SBP, (mmHg)	0.062 (0.056 to 0.066)	0.371	<0.001	0.061 (0.055 to 0.067)	0.352	<0.001	0.061 (0.053 to 0.069)	0.397	<0.001
DBP, (mmHg)	0.082 (0.072 to 0.090)	0.299	<0.001	0.077 (0.066 to 0.089)	0.281	<0.001	0.085 (0.071 to 0.100)	0.329	<0.001
HDL-C, (mg/dl)	0.007 (−0.001 to 0.015)	0.206	0.075	0.012 (0.002 to 0.023)	0.196	0.003	0.012 (−0.008 to 0.014)	0.233	0.561
TGC, (mg/dl)	0.002 (0.001 to 0.003)	0.209	0.001	0.002 (0.001 to 0.003)	0.198	0.002	0.001 (−0.001 to 0.003)	0.234	0.182
FPG, (mg/dl)	0.007 (0.004 to 0.010)	0.210	<0.001	0.005 (0.001 to 0.009)	0.198	0.031	0.010 (0.005 to 0.015)	0.244	0.001
WC, (cm)	−0.004 (−0.012 to 0.004)	0.200	0.315	−0.002 (−0.014 to 0.009)	0.185	0.679	−0.007 (−0.019 to 0.005)	0.226	0.269
*Dependent variable: CAVI*									
SBP, (mmHg)	0.015 (0.013 to 0.017)	0.398	<0.001	0.016 (0.013 to 0.018)	0.430	<0.001	0.014 (0.011 to 0.018)	0.327	<0.001
DBP, (mmHg)	0.020 (0.017 to 0.024)	0.377	<0.001	0.022 (0.017 to 0.026)	0.414	<0.001	0.017 (0.011 to 0.024)	0.302	<0.001
HDL-C, (mg/dl)	−0.001 (−0.004 to 0.002)	0.348	0.578	0.002 (−0.003 to 0.006)	0.382	0.486	−0.002 (−0.007 to 0.002)	0.282	0.301
TGC, (mg/dl)	0.001 (0.001 to 0.001)	0.351	0.002	0.001 (0.001 to 0.001)	0.286	0.002	0.001 (0.001 to 0.001)	0.382	0.289
FPG, (mg/dl)	0.003 (0.002 to 0.005)	0.355	<0.001	0.003 (0.001 to 0.005)	0.387	0.002	0.004 (0.002 to 0.006)	0.289	0.001
WC, (cm)	−0.013 (−0.016 to −0.009)	0.290	<0.001	−0.017 (−0.021 to −0.012)	0.354	<0.001	−0.017 (−0.023 to −0.012)	0.236	<0.001

Multiple linear regression models were used to analyze the associations of components of MetS to baPWV and CAVI values, globally and stratified by gender. Age, height, weight, antihypertensive drugs, lipid-lowering drugs and antidiabetic drugs were adjusted in the regression models. The exception was WC, which was adjusted for age and drug use because of collinearity problems

*MetS* metabolic syndrome; *baPWV* brachial-ankle pulse wave velocity; *CAVI* cardio-ankle vascular index; *CI* confidence interval; *R* ^*2*^ Coefficient of determination; *SBP* systolic blood pressure; *DBP* diastolic blood pressure; *HDL-C* high density lipoprotein cholesterol; *TGC* triglycerides; *FPG* fasting plasma glucose; *WC* waist circumference

The association persisted in the disaggregated analysis in subjects with and without antihypertensive, lipid-lowering, and antidiabetic treatments. The exceptions were the correlation between triglycerides and CAVI and baPWV, which was only seen in the treated subjects, and WC, which was only associated with baPWV in the untreated individuals and with CAVI in the drug treatment group (Additional file 1: Table S1).

The results of the multiple regression analysis of premenopausal and postmenopausal women and males over and under 50 years of age are shown in Additional file 1: Table 2S.

In a logistic regression analysis and after adjusting for potentially influencing variables, the component of MetS with the greatest odds ratio (OR) was high blood pressure for both baPWV ≥ 17.5 m/sec (OR = 6.90, 95 % CI 3.52–13.51) and CAVI ≥ 9 (OR = 2.20, 95 % CI 1.63–1.90) (Table 4).

**Table 4:** Multiple logistic regression analysis of associations between MetS/components and baPWV and CAVI status in males and females

Components MetS	Global (n = 2351)		Males subjects (n = 1450)		Females subjects (n = 901)	
	OR (95 % CI)	p value	OR (95 % CI)	p value	OR (95 % CI)	p value
*Dependent variable: baPWV*						
High BP	6.899 (3.522 to 13.511)	<0.001	7.578 (2.975 to 19.345)	<0.001	5.272 (1.975 to 19.345)	<0.001
High FPG	1.527 (1.123 to 2.077)	0.007	1.290 (0.875 to 1.902)	0.199	2.035 (1.221 to 3.392)	0.006
High TGC	0.745 (0.559 to 0.992)	0.044	0.738 (0.480 to 1.135)	1.116	0.802 (0.534 to 1.205)	0.287
Low HDL-C	1.202 (0.915 to 1.579)	0.186	1.159 (0.811 to 1.656)	0.419	1.329 (0.863 to 2.248)	0.197
High WC	0.965 (0.743 to 1.254)	0.792	0.874 (0.633 to 1.207)	0.414	1.149 (0.675 to 1.953)	0.609
MetS	1.421 (1.062 to 1.902)	0.018	1.512 (1.032 to 2.215)	0.034	1.679 (1.019 to 2.765)	0.042
*Dependent variable: CAVI*						
High BP	2.204 (1.629 to 2.983)	<0.001	2.238 (1.499 to 3.339)	<0.001	2.115 (1.320 to 3.338)	0.002
High FPG	1.368 (1.090 to 1.718)	0.007	1.401 (1.052 to 1.866)	0.021	1.256 (0.857 to 1.842)	0.242
High TGC	0.931 (0.755 to 1.147)	0.500	1.031 (0.768 to 1.384)	0.839	0.888 (0.651 to 1.213)	0.457
Low HDL-C	1.283 (1.043 to 1.579)	0.018	1.374 (1.054 to 1.792)	0.019	1.166 (0.832 to 1.633)	0.372
High WC	0.686 (0.565 to 0.834)	<0.001	0.786 (0.617 to 1.001)	0.051	0.741 (0.511 to 1.075)	0.115
MetS	1.543 (1.235 to 1.927)	<0.001	1.723 (1.286 to 2.307)	<0.001	1.424 (0.990 to 2.048)	0.056

Dependent variable: CAVI and baPWV (values of CAVI ≥9 and baPWV ≥17.5 m/s was considered abnormal)

Multiple logistic regression analysis was used to analyze the associations of MetS status and MetS components with baPWV and CAVI globally and stratified by gender. Age, height, weight, antihypertensive drugs, lipid-lowering drugs and antidiabetic drugs were adjusted in the regression models. The exception was WC, which was adjusted for age and drug use because of collinearity problems

*MetS* metabolic syndrome; *baPWV* brachial-ankle pulse wave velocity; *CAVI* cardio-ankle vascular index; *OR* odds ratio; *CI* confidence interval; *BP* blood pressure; *FPG* fasting plasma glucose; *TGC* triglycerides; *HDL-C* high density lipoprotein cholesterol; WC waist circumference

### Results for subjects with MetS

**Fig. 1:**
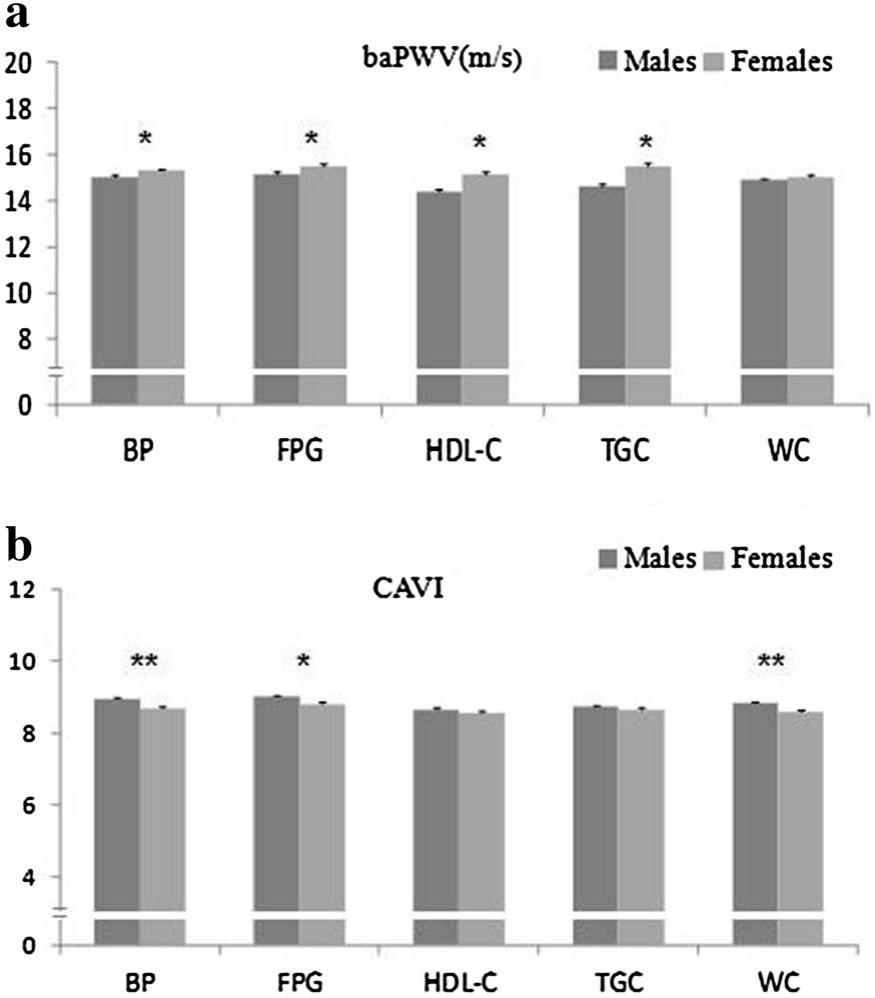
baPWV **a** and CAVI **b** according the MetS components in males and females. Data are given as mean ± standard error. baPWV and CAVI levels were compared using a Student’s t test. Mest criteria: abdominal obesity (n = 1055): WC ≥ 88 in females; ≥ 102 in males. BP (n = 1156): SBP ≥ 130 mmHg and/or DBP ≥ 85 mm Hg or antihypertensive drug treatment. Increase FPG (n = 872): FPG > 100 mg/dL or antidiabetic drug treatment. Reduced HDL-C (n = 667): HDL < 40 mg/dL in males and < 50 mg/dL in females. Increase TGC (n = 692): TGC > 150 mg/dL). *baPWV* brachial-ankle pulse wave velocity, *CAVI* cardio-ankle vascular index, *BP* blood pressure, *FPG* fasting plasma glucose, *HDL-C* high density lipoprotein cholesterol, *TGC* triglycerides, *WC* waist circumference. *p < 0.05 and **p < 0.01 between sexes

Figure 1 shows the mean values corresponding to baPWV and CAVI according to sex and for each of the components of MetS. In the 1220 subjects with MetS, all the MetS components presented higher baPWV values in females except for waist circumference (p > 0.05). However, all the MetS components presented higher CAVI values in males, reaching significant differences in the case of the components related to increased blood pressure, fasting plasma glucose, and waist circumference.

**Fig. 2:**
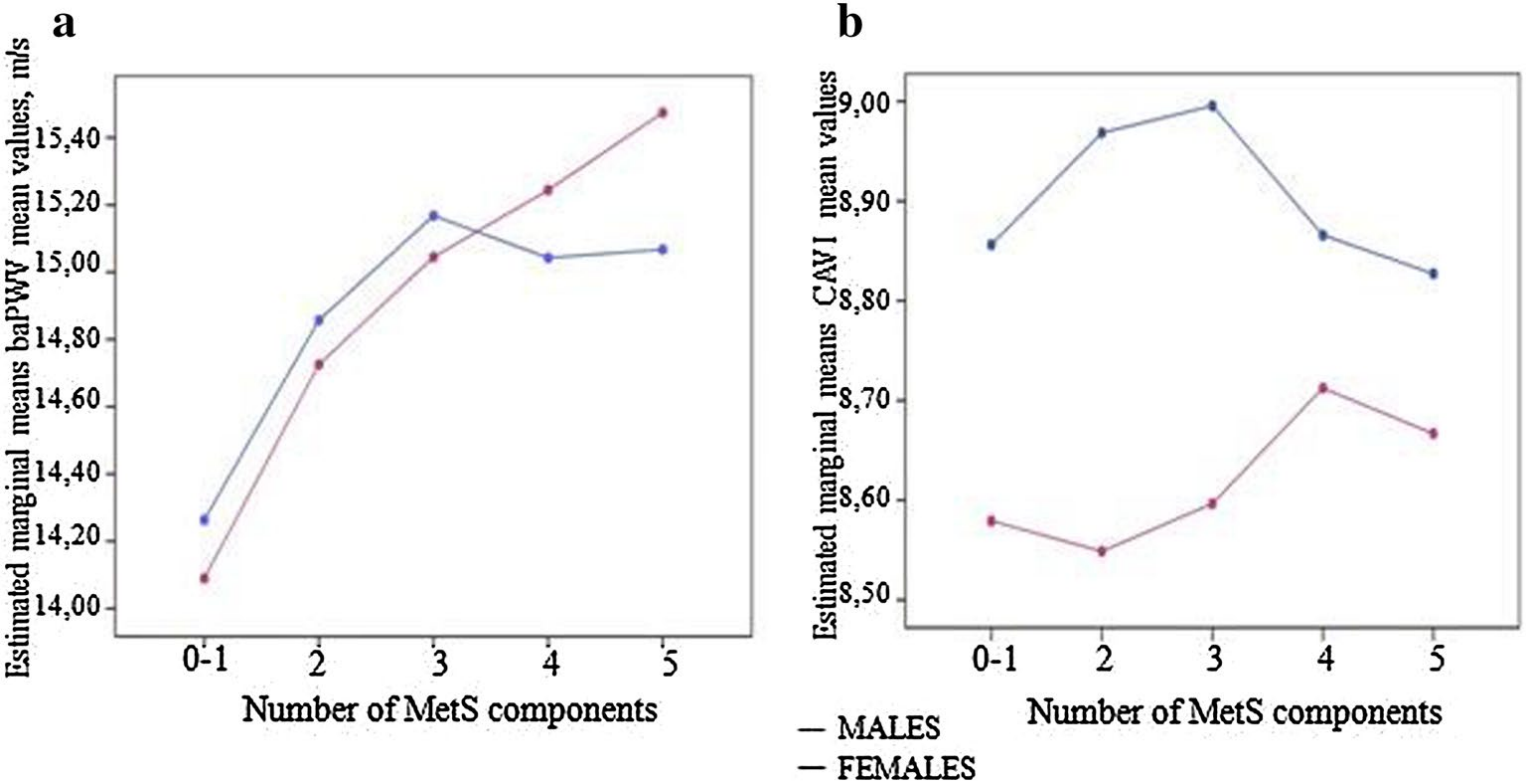
Multivariate analysis (ANCOVA). Brachial-ankle pulse wave velocity (baPWV) values in males and females (**a**) and cardio-ankle vascular index (CAVI) values in males and females (**b**). Values by number of MetS components. Adjusted by age. baPWV differences by number of MetS components in males between 0 and 1 components and 2, 3, and 4 components (p < 0.01); in females between 1 component and 3, 4, and 5 components (p < 0.01). Post-hoc contrasts were performed using a Bonferroni test. *baPWV* brachial-ankle pulse wave velocity, *CAVI* cardio-ankle vascular index, *MetS* metabolic syndrome

Figure 2 shows the change in mean baPWV and CAVI values after age adjustment for increasing number of MetS components. The mean baPWV values in females increased with the number of MetS components. However, in males, the mean baPWV values increased for only up to three MetS components. The mean CAVI values in females increased with two, three, and four MetS components. However, in males, CAVI values increased for up to three MetS components but decreased in groups with four or five components.

**Fig. 3:**
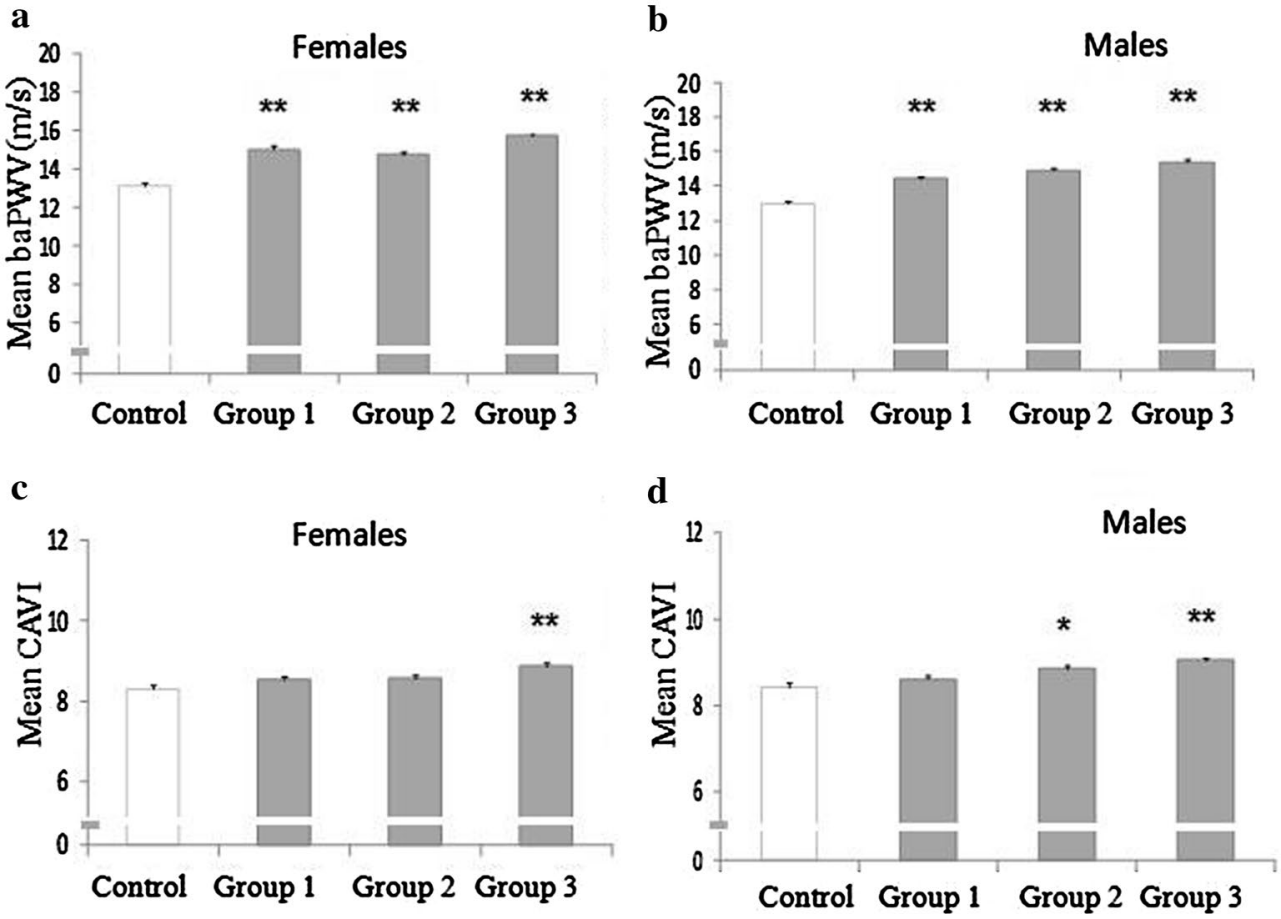
Impact of the specific groups of MetS components on brachial-ankle pulse wave velocity (baPWV) and cardio-ankle vascular index (CAVI) in the different groups. **a** Impact of the group in females on baPWV. **b** Impact of the group in males on baPWV. **c** Impact of the group in females on CAVI. **d** Impact of the group in males on CAVI. Data are given as mean ± standard error. baPWV and CAVI levels were compared using an ANOVA test, followed by post hoc analysis using a Bonferroni test. **p < 0.01 between the different groups and control; *p < 0.05 between the different groups and control. *Group 1* Group MetS-mixed; *Group 2* MetS-dyslipidemia; *Group 3* Group MetS-increased insulin resistance; *Group Control* A group of 175 subjects without MetS, arterial hypertension, FPG or use of antihypertensive, lipid-lowering or antidiabetic drugs was used as control. *baPWV* brachial-ankle pulse wave velocity, *CAVI* cardio-ankle vascular index, *MetS* metabolic syndrome, *FPG* fasting plasma glucose

The results for the mean baPWV and CAVI values according to groups are shown in Additional file 2: Fig. S1 and Fig. 3. The MetS-increased insulin resistance group had the highest CAVI (8.96) and baPWV (15.55 m/sec) in both the global analysis and the sex-based analysis. In the global analysis, all groups of MetS components were associated with higher baPWV and CAVI values compared to the control group (p < 0.01), except for CAVI in the MetS-mixed group.

## Discussion

The results show that in subjects with intermediate cardiovascular risk, MetS and its individual components (except HDL-cholesterol with the two measures and WC with baPWV) are associated with baPWV and CAVI. This association differs according to sex. In males, all MetS components (except HDL-cholesterol with CAVI and WC with baPWV) were associated with both arterial stiffness measures. In females only, SBP, DBP, and FPG were associated with both measures, and WC was associated with CAVI. The arterial stiffness values were highest when the MetS components of increased blood pressure, FPG, and WC occurred simultaneously.

FPG was associated with higher baPWV (15.50 m/sec) and CAVI (9.01) in females and males, respectively. Subjects in this study who had a combination of increased WC, FPG, and BP showed the highest values of both arterial stiffness measures. This finding is Similar to the results of the Framingham Heart Study cohort, where this combination increased the risk of mortality by three-fold [6]. These results suggest that analyzing arterial stiffness in these groups may be helpful for identifying subjects with greater cardiovascular risk [9].

A single study analyzed the relationship of CAVI to MetS and its components. Kawada et al. [24] found no significant association between MetS components and CAVI ≥ 9, with which only sex and age were significantly associated. However, our results suggest that the association of MetS and its components with CAVI is similar to the association with baPWV. These differences are probably due to the study size (144 subjects) and the different ethnic groups of the samples. On the other hand, in a Japanese population, CAVI was shown to be a predictor of cardiovascular events in obese subjects (MetS component) in the Japan Obesity and Metabolic Syndrome study [10]. It should not be forgotten, however, that this is the first study analyzing the association of each MetS component with two arterial stiffness measures and adjustment for different confounding factors in a large simple of subjects at intermediate cardiovascular risk.

The results do not coincide with those published by Satoh et al. [31] for 325 obese Japanese subjects enrolled in the multicenter Japan Obesity and Metabolic Syndrome Study. Their CAVI values were significantly higher in MetS than in non-MetS subjects, and CAVI was closely correlated with the severity of MetS. The discrepancies with our results may have several reasons: the subjects analyzed in this study were older (61.4 versus 49.4 years), and the most prevalent component of MetS in our group was blood pressure (a component less associated with CAVI than other stiffness measures). Other aspects are the different race, cardiovascular risk, and the drugs used for treatment of the different risk factors.

The subjects with arterial hypertension presented a six-fold higher risk of baPWV ≥ 17.5 m/sec, while the risk of CAVI ≥ 9 increased two-fold. These results support several studies [14, 40–42], according to which CAVI as an arterial stiffness measure is independent of blood pressure at the time of measurement. In relation to the rest of the MetS components, the odds ratios were similar for both stiffness measures, although increased waist circumference and low HDL-cholesterol only reached statistical significance with CAVI. This was probably due to the greater percentage of subjects with CAVI ≥ 9.

The MetS components BP and TGC were more common in males, and the WC and HDL cholesterol components were more common in females, while no difference was found in FPG. These results are similar to those reported for a Spanish population in the DARIOS study [43], but different from those reported in Asian populations [5, 7]. Prior studies support these results [7, 25]. Previous studies also analyzed the different behavior of arterial stiffness depending on sex [25, 44]. They found that stiffness was greater in females than males before puberty and increased after menopause. On the other hand, males arterial stiffness increases linearly from puberty, which suggests that women have intrinsically stiffer major arteries than men, but these effects are mitigated by sex steroids during reproductive life [45, 46]. Other factors that may influence these sex differences include height [47], body fat distribution [48], and inflammatory factors [49]. The tool used to assess arterial stiffness may also have an influence. The reason may be that CAVI reflects central and peripheral arterial stiffness [14, 50, 51] and is less influenced by BP values at the time of measurement [14, 40–42]. Arterial stiffness assessed with baPWV, however, is a measure of peripheral arterial stiffness.

The effects of MetS and its components on baPWV are not clear. Several studies on Eastern populations have shown that they are more evident in females than in males [7, 25]. Scuteri et al. [52] reported the impact of MetS on arterial stiffness to be similar in both sexes, and in this study, the number of MetS components associated with arterial stiffness measures was greater in males. However, baPWV and CAVI increased proportionally as the number of components increased in females, which occurred in other studies conducted on Asian populations [7, 8, 23, 25], but not in males. The different characteristics of the populations analyzed and the arterial stiffness measurement based on different parameters may explain these discrepancies.

Each component of MetS has a clear sex-dependent impact on baPWV [52]. As in other studies [7, 47], BP and especially SBP had the greatest association with baPWV and CAVI in our study, in contrast to all other components [25, 52]. Prior studies showed a positive correlation of HDL-C with baPWV in females only [7, 25]. In this study, HDL cholesterol and TGC levels correlated with baPWV in males only, and only TGC correlated with CAVI in males, which is in agreement with the results reported by Weng et al. [25]. However, low HDL cholesterol levels [53] and high TGC levels [54] are predictors of morbidity and mortality from cardiovascular diseases. Further studies analyzing the role of HDL cholesterol and TGC in arterial stiffness are therefore needed.

FPG induces many changes in vascular tissue cells, which may potentially accelerate the atherosclerotic process (mainly in females) [23, 55, 56]. This supports our results found in our study. Abdominal obesity is an essential element in MetS [57] and shows a negative association with CAVI in both sexes. Unlike our work, the association of baPWV with WC has been recorded in both sexes in Asian populations [23, 25]. These differences are related to the adjustment variables used.

The greater decrease in CAVI among the subjects with more MetS components could be due to the lesser influence of blood pressure on CAVI [14, 40–42]. Another possible explanation is the greater percentage of subjects receiving drug treatment in the groups with 4 or 5 MetS components. Thus, while 44 % of the subjects in the group with 0 or 1 component were receiving drugs for hypertension, diabetes, or dyslipidemia, the corresponding percentages were 77 and 88 % among the subjects with 4 and 5 MetS components, respectively.

Our results suggest that in Caucasian subjects with intermediate cardiovascular risk, arterial stiffness is associated with the MetS components, except HDL-cholesterol for baPWV and CAVI and WC for baPWV except for HDL-cholesterol with respect to both stiffness parameters and waist circumference with respect to baPWV. However, the association of triglycerides and HDL-cholesterol was only observed in males. These differences could have clinical relevance and may help to explain the discrepancies in cardiovascular risk between sexes. The results suggest that the treatment of hypertriglyceridemia could improve arterial stiffness, particularly in males with MetS.

The *main limitation* of our study is its cross-sectional design, which cannot establish causal relations or the direction of the impact of MetS on CAVI and baPWV. An additional limitation is the impact on arterial stiffness of drugs for treating specific MetS components such as blood glucose, dyslipidemia, and blood pressure. However, we did try to mitigate this effect by including them as adjustment variables in the regression analysis.

## Conclusions

MetS and most of its individual components (except HDL-cholesterol for baPWV and CAVI and WC for baPWV) were associated with baPWV and CAVI. However, there were differences between sex in the association of MetS and its components with baPWV and CAVI. The data suggest a greater association of CAVI and baPWV values with MetS components in males than in females and indicate greater arterial stiffness upon simultaneously elevated blood pressure, fasting plasma glucose, and waist circumference. Therefore, the determination of arterial stiffness based on CAVI and baPWV may be useful for evaluating the cardiovascular risk of MetS and its components in Caucasian adults with intermediate cardiovascular risk.

## Additional files

**Additional file 1: Table S1.** Associations of MetS components with baPWV and CAVI values treatment in subjects with and without drug treatment. **Table S2:** Associations of MetS components with baPWV and CAVI values premenopausal and postmenopausal females in older males and younger than 50 years.

**Additional file 2: Figure S1.** Impact of the specific groups of MetS components on brachial-ankle pulse wave velocity (baPWV) and cardio-ankle vascular index (CAVI) in the different groups. **a** Impact of the group on baPWV. **b** Impact of the group i on baPWV. Data are given as mean ± standard error. baPWV and CAVI levels were compared using an ANOVA test, followed by post hoc analysis using a Bonferroni test. **p < 0.01 between the different groups and control; *p < 0.05 between the different groups and control. baPWV brachial-ankle pulse wave velocity; CAVI cardio-ankle vascular index; MetS metabolic syndrome. Group 1: Group MetS-mixed. Group 2: MetS-dyslipidemia. Group 3: Group MetS-increased insulin resistance. Group Control: A group of 175 subjects without MetS, arterial hypertension, fasting plasma glucose or use of antihypertensive, lipid-lowering or antidiabetic drugs was used as control.

## Abbreviations

baPWV: brachial-ankle pulse wave velocity
BMI: body mass index
BP: blood pressure
CAVI: cardio-ankle vascular index
DBP: diastolic blood pressure
FPG: fasting plasma glucose
HDL-C: high-density lipoprotein cholesterol
MetS: metabolic syndrome
MARK: MediAte RisK
OR: odds ratio
SBP: systolic blood pressure
TGC: triglycerides
WC: waist circumference
