# Supplementary material for: Association of metabolic syndrome and its components with arterial stiffness in Caucasian subjects of the MARK study: a cross-sectional trial
Source: Cardiovasc Diabetol. 2016 Oct 24;15:148. doi: 10.1186/s12933-016-0465-7 (PMC5078926; doi:10.1186/s12933-016-0465-7)
Supplement: Supplementary file 1 — Additional file 1: Table S1. Associations of MetS components with baPWV and CAVI values treatment in subjects with and without drug treatment. Table S2: Associations of MetS components with baPWV and CAVI values premenopausal and postmenopausal females in older males and younger than 50 years. [file 12933_2016_465_MOESM1_ESM.doc]

**Table 1S: Associations of MetS components with baPWV and CAVI values** treatment in subjects with and without drug treatment.

| **Components MetS** | | | |  | **Without treatment (n=784)** | | | | |  | | | **With treatment (n=1567)** | | | | | | |
| --- | --- | --- | --- | --- | --- | --- | --- | --- | --- | --- | --- | --- | --- | --- | --- | --- | --- | --- | --- |
|  | | | | **β (95%CI)** | | **R^2^** | | **p value** | | **β (95%CI)** | | | | **R^2^** | | **p value** | | | |
|  | **Dependent variable: baPWV** | | | | | | | | | | | | | | | | | |  |
| SBP, (mmHg) | | | 0.057 (0.050 to 0.065) | | | | 0.408 | | <0.001 | | | 0.063 (0.057to0.069) | | | 0.317 | | <0.001 | | |
| DBP, (mmHg) | | | 0.070 (0.057 to 0.083) | | | | 0.323 | | <0.001 | | | 0.082 (0.070to0.094) | | | 0.329 | | <0.001 | | |
| HDL-C, (mg/dl) | | | 0.005 (-0.006 to 0.017) | | | | 0.228 | | 0.367 | | | 0.005 (-0.005to0.023) | | | 0.015 | | 0.297 | | |
| TGC, (mg/dl) | | | 0.001 (-0.001 to 0.003) | | | | 0.229 | | 0.120 | | | 0.003 (0.001to0.003) | | | 0.157 | | 0.003 | | |
| FPG, (mg/dl) | | | 0.007 (0.004 to 0.010) | | | | 0.218 | | <0.001 | | | 0.005 (0.001to0.009) | | | 0.172 | | 0.031 | | |
| WC, (cm) | | | 0.016 (0.004 to 0.029) | | | | 0.233 | | 0.012 | | | -0.010(-0.021to0.001) | | | 0.140 | | 0.055 | | |
|  | **Dependent variable: CAVI** | | | | | | | | | | | | | | | | | |  |
| SBP, (mmHg) | | 0.015 (0.011 to 0.019) | | | | | 0.418 | | <0.001 | | 0.015 (0.013 to 0.018) | | | | 0.360 | | | <0.001 | |
| DBP, (mmHg) | | 0.016 (0.010 to 0.023) | | | | | 0.389 | | <0.001 | | 0.020 (0.015 to 0.025) | | | | 0.338 | | | <0.001 | |
| HDL-C, (mg/dl) | | 0.005 (-0.005 to 0.005) | | | | | 0.368 | | 0.990 | | -0.002 (-0.006 to 0.002) | | | | 0.311 | | | 0.226 | |
| TGC,(mg/dl) | | 0.001 (-0.001 to 0.001) | | | | | 0.369 | | 0.172 | | 0.001 (0.001 to 0.001) | | | | 0.314 | | | 0.005 | |
| FPG, (mg/dl) | | 0.004 (0.001 to 0.007) | | | | | 0.374 | | 0.007 | | 0.005 (0.004 to 0.006) | | | | 0.335 | | | <0.001 | |
| WC, (cm) | | -0.005 (-0.011 to 0.000) | | | | | 0.320 | | 0.072 | | -0.015 (-0.020to -0.011) | | | | 0. 251 | | | <0.001 | |

Multiple linear regression analysis were used to analyze the associations of MetS status and METS components to baPWV and CAVI globally and stratified by gender. Age, height, weight, antihypertensive drugs, lipid-lowering drugs and antidiabetic drugs were adjusted in the regression models. The exception was WC, which was adjusted for age and drug use because of collinearity problems.

MetS metabolic syndrome; baPWV brachial-ankle pulse wave velocity; CAVI cardio-ankle vascular index; CI conﬁdence interval; R^2^ Coefficient of determination; SBP systolic blood pressure; DBP diastolic blood pressure; HDL-C high density lipoprotein cholesterol; TGC triglycerides; FPG fasting plasma glucose; WC waist circumference.

**Table 2S: Associations of MetS components with baPWV and CAVI values premenopausal and postmenopausal females in older males and younger than 50 years.**

| **Components MetS** |  | **Premenopausal (n=53)** | | | | | | |  | | | | **Males < 50 years (n=163)** | | | | | | | | | | |  | | |
| --- | --- | --- | --- | --- | --- | --- | --- | --- | --- | --- | --- | --- | --- | --- | --- | --- | --- | --- | --- | --- | --- | --- | --- | --- | --- | --- |
|  | **β (95%CI)** | | **R^2^** | | | **p value** | | | **β (95%CI)** | | | | | **R^2^** | | | | **p value** | | | | | |  | | |
| **Dependent variable: baPWV** | | | | | | | | | | | | | | | | | | | | | | | |  | | |
| SBP, (mmHg) | 0.040 (0.015 to 0.075) | | | | 0.342 | | | <0.004 | | | 0.057 (0.040 to 0.074) | | | | | 0.241 | | | | | <0.001 | | | | | |
| DBP, (mmHg) | 0.061 (0.015 to 0.107) | | | | 0.316 | | | 0.011 | | | 0.063 (0.037 to 0.090) | | | | | 0.150 | | | | | <0.001 | | | | | |
| HDL-C, (mg/dl) | 0.008 (-0.023 to 0.017) | | | | 0.039 | | | 0.605 | | | 0.008 (-0.023 to 0.033) | | | | | 0.711 | | | | | 0.367 | | | | | |
| TGC, (mg/dl) | 0.003 (-0.010 to 0.003) | | | | 0.229 | | | 0.293 | | | 0.001 (0.001 to 0.003) | | | | | 0.053 | | | | | 0.050 | | | | | |
| FPG, (mg/dl) | 0.005 (-0.021 to 0.010) | | | | 0.218 | | | 0.490 | | | 0.008 (-0.001 to 0.016) | | | | | 0.049 | | | | | 0.071 | | | | | |
| WC, (cm) | -0.015 (-0.055 to 0.026) | | | | 0.223 | | | 0.472 | | | -0.002 (-0.023 to 0.020) | | | | | 0.036 | | | | | 0.884 | | | | | |
| **Dependent variable: CAVI** | | | | | | | | | | | | | | | | | | | | | | |  | | | |
| SBP, (mmHg) | 0.011 (-0.005 to 0.027) | | | | 0.260 | | | 0.173 | | | 0.018 (0.009 to 0.027) | | | | | 0.273 | | | | | | <0.001 | | | | |
| DBP, (mmHg) | 0.014 (-0.010 to 0.038) | | | | 0.252 | | | 0.240 | | | 0.021 (0.007 to 0.035) | | | | | 0.247 | | | | | | 0.004 | | | | |
| HDL-C, (mg/dl) | 0.001 (-0.015 to 0.015) | | | | 0.228 | | | 0.975 | | | 0.003 (-0.012 to 0.017) | | | | | 0.205 | | | | | | 0.725 | | | | |
| TGC, (mg/dl) | 0.001 (-0.004 to 0.002) | | | | 0.234 | | | 0.554 | | | 0.001 (-0.001 to 0.001) | | | | | 0.214 | | | | | | 0.168 | | | | |
| FPG, (mg/dl) | -0.002 (-0.009 to 0.006) | | | | 0.231 | | | 0.667 | | | 0.003 (-0.001 to 0.007) | | | | | 0.216 | | | | | | 0.124 | | | | |
| WC, (cm) | -0.022 (-0.043 to -0.001) | | | | 0.146 | | | 0.039 | | | -0.029 (-0.040to -0.018) | | | | | 0.188 | | | | | | <0.001 | | | | |
| **Components MetS** |  | **Postmenopausal (n=849)** | | | | | | |  | | | | **Males> 50 years (n=1287)** | | | | | | | | | |  | | | |
|  | **β (95%CI)** | | **R^2^** | | | **p value** | | | **β (95%CI)** | | | | | **R^2^** | | | **p value** | | | | | |  | | | |
| **Dependent variable: baPWV** | | | | | | | | | | | | | | | | | | | | | | |  | | | |
| SBP, (mmHg) | 0.062 (0.054 to 0.070) | | | 0.368 | | | <0.001 | | | | | 0.061 (0.054 to 0.068) | | | 0.304 | | | | <0.001 | | | | | | |  |
| DBP, (mmHg) | 0.088 (0.073 to 0.104) | | | 0.295 | | | <0.001 | | | | | 0.082 (0.069 to 0.095) | | | 0.233 | | | | <0.001 | | | | | | |  |
| HDL-C, (mg/dl) | 0.003 (-0.008 to 0.015) | | | 0.191 | | | 0.579 | | | | | 0.013 (0.002 to 0.025) | | | 0.138 | | | | 0.023 | | | | | | |  |
| TGC, (mg/dl) | 0.001 (-0.001 to 0.004) | | | 0.193 | | | 0.156 | | | | | 0.002 (0.001 to 0.004) | | | 0.139 | | | | 0.004 | | | | | | |  |
| FPG, (mg/dl) | 0.011 (0.005 to 0.016) | | | 0.204 | | | <0.001 | | | | | 0.004 (-0.001 to 0.009) | | | 0.136 | | | | 0.089 | | | | | | |  |
| WC, (cm) | -0.007(-0.020 to 0.006) | | | 0.183 | | | 0.281 | | | | | -0.002(-0.016 to 0.011) | | | 0.125 | | | | 0.708 | | | | | | |  |
| **Dependent variable: CAVI** | | | | | | | | | | | | | | | | | | | | | | | | |  | |
| SBP, (mmHg) | 0.014 (0.011 to 0.018) | | | 0.273 | | | <0.001 | | | 0.015 (0.012 to 0.018) | | | | | 0.321 | | | | | <0.001 | | | | | |  |
| DBP, (mmHg) | 0.017 (0.011 to 0.024) | | | 0.244 | | | <0.001 | | | 0.021 (0.016 to 0.027) | | | | | 0.301 | | | | | <0.001 | | | | | |  |
| HDL-C, (mg/dl) | -0.002 (-0.007 to0.003) | | | 0.222 | | | 0.364 | | | -0.002 (-0.003 to 0.006) | | | | | 0.265 | | | | | 0.471 | | | | | |  |
| TGC, (mg/dl) | 0.001 (-0.001 to 0.001) | | | 0.222 | | | 0.369 | | | 0.001 (0.001 to 0.002) | | | | | 0.270 | | | | | 0.003 | | | | | |  |
| FPG, (mg/dl) | 0.004 (0.001 to 0.006) | | | 0.231 | | | 0.002 | | | 0.003 (0.001 to 0.005) | | | | | 0.270 | | | | | 0.002 | | | | | |  |
| WC, (cm) | -0.017(-0.023to -0.011) | | | 0.177 | | | <0.001 | | | -0.014 (-0.019 to -0.009) | | | | | 0. 234 | | | | | <0.001 | | | | | |  |

Multiple linear regression analysis were used to analyze the associations of MetS status and MetS components to baPWV globally and stratified by gender. Age, height, weight, antihypertensive drugs, lipid-lowering drugs and antidiabetic drugs were adjusted in the regression models. The exception was WC, which was adjusted for age and drug use because of collinearity problems.

MetS metabolic syndrome; baPWV brachial-ankle pulse wave velocity; CAVI cardio-ankle vascular index; CI conﬁdence interval; R^2^ Coefficient of determination; SBP systolic blood pressure; DBP diastolic blood pressure; HDL-C high density lipoprotein cholesterol; TGC triglycerides; FPG fasting plasma glucose; WC waist circumference.
