# Supplementary figures and images for: Association of metabolic syndrome and its components with arterial stiffness in Caucasian subjects of the MARK study: a cross-sectional trial
Source: Cardiovasc Diabetol. 2016 Oct 24;15:148. doi: 10.1186/s12933-016-0465-7 (PMC5078926; doi:10.1186/s12933-016-0465-7)

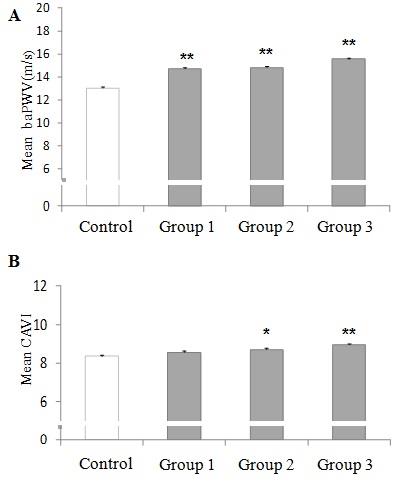

Supplement: Supplementary file 2 — Additional file 2: Figure S1. Impact of the specific groups of MetS components on brachial-ankle pulse wave velocity (baPWV) and cardio-ankle vascular index (CAVI) in the different groups. a Impact of the group on baPWV. b Impact of the group i on baPWV. Data are given as mean ± standard error. baPWV and CAVI levels were compared using an ANOVA test, followed by post hoc analysis using a Bonferroni test. **p < 0.01 between the different groups and control; *p < 0.05 between the different groups and control. baPWV brachial-ankle pulse wave velocity; CAVI cardio-ankle vascular index; MetS metabolic syndrome. Group 1: Group MetS-mixed. Group 2: MetS-dyslipidemia. Group 3: Group MetS-increased insulin resistance. Group Control: A group of 175 subjects without MetS, arterial hypertension, fasting plasma glucose or use of antihypertensive, lipid-lowering or antidiabetic drugs was used as control. [file 12933_2016_465_MOESM2_ESM.jpg]
